# The *NF1* gene revisited – from bench to bedside

**DOI:** 10.18632/oncotarget.2194

**Published:** 2014-07-07

**Authors:** Yoon-Sim Yap, John R McPherson, Choon-Kiat Ong, Steven G Rozen, Bin-Tean Teh, Ann SG Lee, David F Callen

**Affiliations:** ^1^ Division of Medical Oncology, National Cancer Centre Singapore, Singapore; ^2^ Faculty of Health Sciences, School of Medicine, University of Adelaide, Australia; ^3^ Centre for Computational Biology, Cancer and Stem Cell Biology Program, Duke-National University of Singapore Graduate Medical School, Singapore; ^4^ Laboratory of Cancer Epigenome, Division of Medical Sciences, National Cancer Centre Singapore, Singapore; ^5^ Division of Cancer and Stem Cell Biology, Duke–National University of Singapore Graduate Medical School, Singapore; ^6^ Cancer Science Institute of Singapore, National University of Singapore, Singapore; ^7^ Laboratory of Molecular Oncology, Division of Medical Sciences, National Cancer Centre Singapore, Singapore; ^8^ Office of Clinical & Academic Faculty Affairs, Duke-National University of Singapore Graduate Medical School, Singapore; ^9^ Department of Physiology, Yong Loo Lin School of Medicine, National University of Singapore, Singapore; ^10^ Cancer Therapeutics Laboratory, Centre for Personalised Cancer Medicine, University of Adelaide, Australia

**Keywords:** neurofibromatosis type 1, NF1, neurofibromin, cancer

## Abstract

Neurofibromatosis type 1 (NF1) is a relatively common tumour predisposition syndrome related to germline aberrations of *NF1*, a tumour suppressor gene. The gene product neurofibromin is a negative regulator of the Ras cellular proliferation pathway, and also exerts tumour suppression via other mechanisms.

Recent next-generation sequencing projects have revealed somatic *NF1* aberrations in various sporadic tumours. *NF1* plays a critical role in a wide range of tumours. *NF1* alterations appear to be associated with resistance to therapy and adverse outcomes in several tumour types.

Identification of a patient's germline or somatic *NF1* aberrations can be challenging, as *NF1* is one of the largest human genes, with a myriad of possible mutations. Epigenetic factors may also contribute to inadequate levels of neurofibromin in cancer cells.

Clinical trials of *NF1*-based therapeutic approaches are currently limited. Preclinical studies on neurofibromin-deficient malignancies have mainly been on malignant peripheral nerve sheath tumour cell lines or xenografts derived from NF1 patients. However, the emerging recognition of the role of *NF1* in sporadic cancers may lead to the development of *NF1*-based treatments for other tumour types. Improved understanding of the implications of *NF1* aberrations is critical for the development of novel therapeutic strategies.

## INTRODUCTION

Neurofibromatosis type 1, also known as NF1 or von Recklinghausen's disease, is a tumour predisposition syndrome characterized by the development of multiple neurofibromas, café-au-lait spots and Lisch nodules. Initially described by Professor Von Recklinghausen, a German pathologist back in 1882, NF1 is one of the most common genetic disorders worldwide [1, 2]. The *NF1* gene is a classic tumour suppressor gene on chromosome 17. Its product neurofibromin is an important negative regulator of Ras cellular proliferation pathways [3-7]. Individuals with NF1 are at increased risk of developing various tumours, including malignant peripheral nerve sheath tumour (MPNST), phaeochromocytoma, leukaemia, glioma, rhabdomyosarcoma and breast cancer [8, 9]. Neurofibromatosis type 1 or NF1 is distinct from neurofibromatosis type 2 (NF2), which is less common. NF2 syndrome is related to mutations in *NF2* on chromosome 22, with a different spectrum of tumours, notably schwannomas, meningiomas and ependymomas [10].

More recently, somatic *NF1* aberrations have been increasingly reported in various sporadic tumours, including brain, lung, breast, ovarian tumours and melanomas. Significant challenges remain in the detection of both germline and somatic aberrations. A better understanding of the implications of these aberrations is critical for the improvement of treatment outcomes of tumours with *NF1* aberrations.

### NF1 syndrome

NF1 is a relatively common genetic condition, with an incidence of approximately 1 in 2,000 to 1 in 5,000 individuals worldwide [2]. Although it is an autosomal dominant genetic disorder, approximately half of the cases have no family history, with the condition arising from sporadic mutations of the *NF1* gene. The germline *NF1* mutation rate is ten-fold higher than that observed in other inherited disease genes, with estimates from 1/7,800 to 1/23,000 gametes [2, 11].

The condition has 100% penetrance but its degree of expression varies considerably, even within the same family with the identical mutation [12]. NF1 is diagnosed clinically for most patients, with genetic testing reserved for equivocal cases or in the context of research studies. The National Institutes of Health (NIH) diagnostic criteria stipulate that at least 2 of the criteria in Table 1 must be fulfilled to make the clinical diagnosis of NF1 [13].

Loss-of-function mutations in the *NF1* gene can also lead to the development of a wide range of abnormalities in the cardiovascular, musculoskeletal and nervous systems, in addition to the predisposition to benign and malignant tumours. Hypertension, vasculopathy, valvular dysfunction, skeletal anomalies, dysmorphic features, osteoprorosis, cognitive impairment and epilepsy may occur as part of the NF1 syndrome [14].

**Table 1:** National Institutes of Health (NIH) diagnostic criteria for neurofibromatosis type 1 (NF1)

Six or more café-au-lait macules >5mm in greatest diameter in prepubertal individuals, and >15mm in postpubertal individuals Two or more neurofibromas of any type or one plexiform neurofibroma Freckling in the axillary or inguinal regions Optic glioma Two or more iris hamartoma (Lisch nodules) Distinctive bony lesion such as sphenoid dysplasia, or thinning of the long bone cortex with or without pseudoarthrosis A first-degree relative (parent, sibling or offspring) with NF1 based on the above criteria

The NF1 phenotype is highly variable, ranging from a very mild manifestation of the disease in certain individuals, to a very severe form in some others [12]. In general, there is no definite correlation between a particular alteration and phenotype. Exceptions include deletion of the entire *NF1* gene which is associated with a severe form of the disease [15], a recurrently ascertained 3-bp in-frame deletion of exon 17 (c.2970-2972 delAAT) that is associated with the typical pigmentary NF1 features but without cutaneous or surface plexiform neurofibromas [16], and duplication of the *NF1* locus which usually leads to intellectual impairment and epilepsy without the other NF1 features [17, 18]. Intra- and interfamilial variation in severity of the phenotype suggests that expression of the same genotype may be influenced by epigenetic or environmental factors [12, 19]. Females with NF1 often experience an exacerbation of the condition following pregnancy, possibly related to changes in the hormonal milieu [20].

This overview will focus on mainly the oncological aspects of *NF1* aberrations, given the recent discovery of somatic *NF1* aberrations in various cancers in individuals without germline NF1.

### Biology of NF1 and neurofibromin

Identified and cloned in 1990, the *NF1* gene is located at chromosome 17q11.2 [4, 21], and is one of the largest genes in the human genome, with 60 exons spanning over 350kb of genomic DNA [4, 22]. Another distinctive feature of the gene is the presence of 3 genes in intron 27b on the antisense strand: OMGP (oligodendrocyte-myelin glycoprotein), a membrane glycoprotein, and EVI2A and EVI2B (ecotropic viral integration sites), which are involved in the development of mouse leukemia [23, 24].

*NF1* encodes the protein neurofibromin, which has an estimated molecular mass of 327kDa and consists of 2818 amino acids. Neurofibromin is ubiquitously expressed, but most highly in the central nervous system, especially in neurons, astrocytes, oligodendrocytes and Schwann cells [25]. As might be expected for such a large gene, alternate exons, splice variants and alternate start sites have been reported. The major reported functional isoforms are derived from the insertion of extra exons that preserve the open reading frame and show tissue restricted expression.

The two major isoforms are neurofibromin types I and II. Neurofibromin type I is expressed predominantly in the brain, and has significant Ras regulatory activity. Neurofibromin type II, also known as GRD2 (domain II-related GAP) is the product of the insertion of exon 23a. In contrast to neurofibromin type 1, it has limited GAP regulatory function [26, 27]. It is expressed mainly in Schwann cells, and is essential for learning and memory in mouse models. In studies on sporadic colon, ovarian and breast cancers as well as gastric cancer cell lines, expression of the type I isoform relative to type II isoform is increased in tumour samples compared to normal tissue [28-31].

Information on other neurofibromin isoforms is limited. Neurofibromin types III and IV, which contain exon 48a and both exons 23a and 48a respectively, are expressed in mainly cardiac and skeletal muscles. They appear to be essential for normal muscle and cardiac development [32, 33]. Apart from neurofibromin types I-IV, two other isoforms have been described. An isoform which contains exon 9a is expressed mainly in neurons of the forebrain, and may be involved in memory and learning mechanisms [34, 35]. Another isoform has alternative exon 10a-2 inserted, introducing a transmembrane domain. The function of this variant, which is observed in a majority of human tissues, is unclear, but may perform a housekeeping function in intracellular membranes [36].

### Roles of NF1 and neurofibromin in tumour suppression

*NF1* is considered a classical tumour suppressor gene, with both copies of the *NF1* gene reported to be inactivated in benign and malignant tumours in NF1 patients [37-39]. The first hit is inherited or acquired as a germline mutation, and the second hit occurs from a somatic event. Loss of heterozygosity (LOH) due to large somatic rearrangements, deletions and somatic recombination may affect the wild-type *NF1* allele. This can also potentially affect other genes on chromosome 17, which include the tumour suppressor protein *p53* at 17p13.2, human epidermal growth factor receptor 2 (*HER2*) at 17q21.1, topoisomerase II alpha (*TOP2A*)(17q21.1), signal transducer and activator of transcription 3 (*STAT3*)(17q21.2) and breast cancer gene 1 (*BRCA1*)(17q21.2) [40].

Various *Nf1*^+/−^ mouse models show predisposition to tumour formation, including phaeochromocytomas, leukaemias and malignant peripheral nerve sheath tumours (MPNST), similar to the spectrum of NF1-associated malignancies observed in human counterparts [41-43].

**Figure 1:**
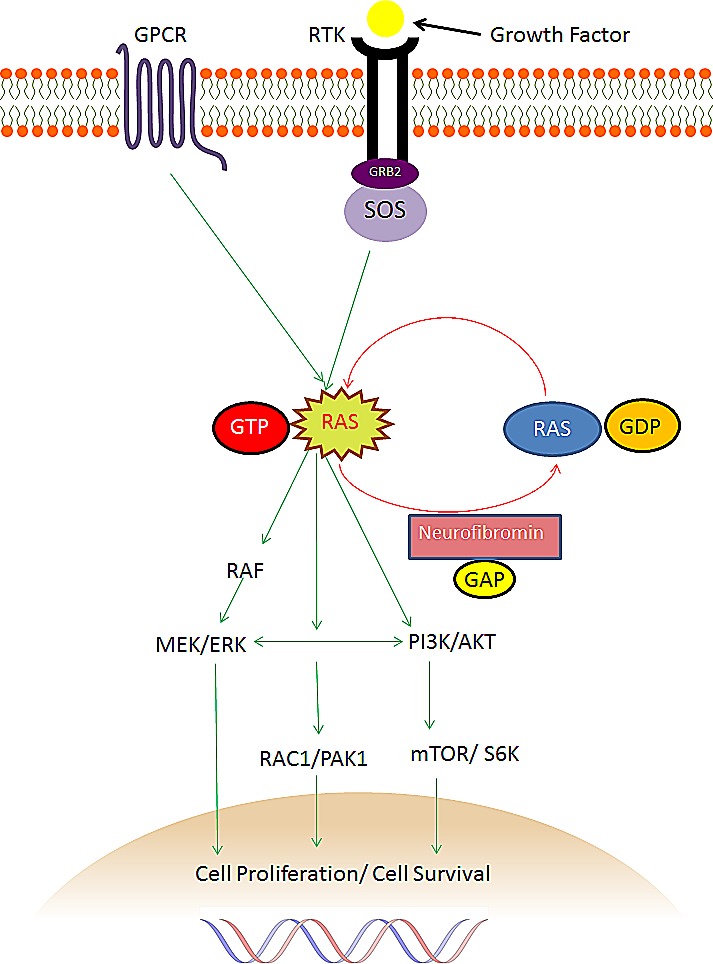
The role of NF1 and neurofibromin in the Ras pathway G-protein coupled receptors (GPCRs) and receptor tyrosine kinases (RTKs), when activated by ligand, promote guanine nucleotide exchange to form activated Ras-GTP complex. Neurofibromin inactivates Ras by accelerating the conversion of active Ras-GTP to inactive GDP-bound Ras with its Ras-GTPase activity. Consequently, neurofibromin suppresses activation of the downstream effectors of Ras, including PI3K, Akt, mTOR, S6 kinase and RAF, MEK, ERK as well as RAC1 and PAK1. RTKs=receptor tyrosine kinases. Grb2=growth factor receptor bound 2. SOS=mammalian homolog of the Drosophila son of sevenless. RAS=rat sarcoma viral oncogene homologue. GDP=guanosine diphosphate. GTP=guanosine triphosphate. RAF=murine sarcoma viral oncogene homologue. MEK=MAPK-ERK kinase. PI3K=phosphatidylinositol-3–kinase. AKT=V-akt murine thymoma viral oncogene homologue 1. mTOR=mammalian target of rapamycin. Rac1=Ras-related C3 botulinum toxin substrate 1. PAK1=P21-Activated Kinase.

The tumour suppressor function of neurofibromin is largely attributed to a small central region which comprises 360 amino acids encoded by exons 20-27a. This critical region has marked structural and sequence similarity to ras-guanosine-triphosphate(GTP)ase activation proteins (GAPs) and is known as the GAP-related domain (GRD). GAPs inactivate Ras by accelerating the conversion of active Ras-GTP to its inactive guanosine diphosphate (GDP)-bound form. The downregulation of oncogene Ras by neurofibromin prevents the downstream activation of mitogen-activated protein kinase (MAPK) and the PI3K/Akt/mTOR (mammalian target of rapamycin) cell proliferation and differentiation pathways, as demonstrated in Figure 1 below [3, 5-7, 44].

The Ras-GAP function of neurofibromin may be enhanced by protein kinase C (PKC) phosphorylation of the cystein-serine rich domain (CSRD) of the neurofibromin domain encoded by exons 11-17. The clustering of missense mutations in these regions among NF1 patients indicate the importance of PKC phosphorylation in sustaining normal neurofibromin function [14, 45-48].

Neurofibromin has also been demonstrated to bind to caveolin-1 (Cav-1), a membrane protein which regulates signalling molecules such as p21^ras^, protein kinase C and growth factor receptors. Formation of the neurofibromin-Cav-1 complex may lead to inactivation of p21^ras^-GTP and modulation of the p21^ras^/MAPK, PI3K/Akt pathways, controlling cell proliferation and differentiation [14, 49].

Apart from downregulation of Ras via the homology to GAPs, there are several other postulated mechanisms for the tumour suppressor function of neurofibromin (Table 2).

**Table 2:** Mechanisms of Tumour Suppression by Neurofibromin

Mechanisms of Tumour Suppression Reported
Downregulation of Ras Positive regulation of adenyl cyclase (AC) Pro-apoptotic effect (ras-dependent and ras-independent) Regulation of cell adhesion and motility Suppression of epithelial mesenchymal transition (EMT) Suppression of heat shock factor (HSF)

Neurofibromin is a positive regulator of the enzyme adenylyl cyclase (AC), which generates intracellular cyclic AMP (cAMP). cAMP-dependent signaling appears to be important in learning and memory, but also provides a possible mechanism for tumour suppressor function as it regulates Ras activity [50, 51]. Increased cAMP leads to activation of Rap1, an anti-mitogenic RAS pathway antagonist, which can result in inhibition of RAF activation in astrocytes [52, 53]. cAMP-mediated regulation of MAPK may have differential effects in different tissues; the mechanisms of cAMP-mediated tumorigenesis in tissues outside the nervous system have not yet been elucidated.

Neurofibromin has also been reported to exert tumour suppressor function via a proapoptotic effect by Ras-dependent and Ras-independent pathways. *Nf1*^−/−^, *Nf1*^+/−^, and *Nf1*^+/+^ mouse embryonic fibroblasts (MEFs) exhibited gene-dosage-related resistance to apoptosis. Neurofibromin-deficient MEFs and human NF1 malignant peripheral nerve sheath tumour (MPNST) cells were more resistant to apoptosis than neurofibromin-expressing MEFs and schwannoma cells. Administration of farnesylthiosalicyic acid (FTS), a Ras inhibitor, increased apoptosis of the neurofibromin-deficient SV40 MEFs and MPNST cells, indicating dependence on the Ras pathway. However, the resistance of neurofibromin-deficient SV40 MEFs and MPNST cells to staurosporine (protein kinase C inhibitor which induces apoptosis), UV irradiation, and vincristine was independent of Ras and cAMP, as demonstrated by the inability of Ras inhibitors or agents that elevate cAMP levels to overcome the resistance. Expression levels of key apoptotic components such as Bcl-2 family proteins, caspases and the X-linked inhibitor of apoptosis (XIAP) were similar in neurofibromin-expressing and neurofibromin-deficient MEFs. The exact mechanism of the Ras-independent proapoptotic effects of neurofibromin remains unclear [54].

The role of neurofibromin in cell motility is important not only for the functioning in neurons, but may also contribute to its tumour suppressor function. Neurofibromin regulates the dynamics and reorganisation of actin filaments via the Rho-ROCK-LIMK2-cofillin pathway, and may be involved in adhesion and signalling at neuronal synapses through its interaction (via its GRD and C-terminal domains) with the transmembrane heparin sulphate proteoglycan syndecan. Lack of neurofibromin triggers the Rho-ROCK-LIMK2-cofilin pathway to alter the organization of actin cytoskeleton, promoting cell motility, invasiveness, and cell-cell adhesion, resulting in the formation of large cell aggregates. This may lead to the formation of multiple neurofibromas in NF1 patients, which consist of aggregates of various cell types, including Schwann cells, fibroblasts, endothelial cells and mast cells on a background of excessive extracellular matrix deposition [55, 56].

Another mechanism of tumour suppression by neurofibromin relates to its association with the N-terminal of focal adhesion kinase (FAK), a protein localised at contact sites of cells with extracellular matrix known as focal adhesions. This interaction helps to regulate cellular events including adhesion, proliferation, motility, cellular migration and survival. *Nf1^+/+^* mouse embryonic fibroblast (MEF) cells exhibited less growth under serum deprivation conditions with reduced adherence on collagen and fibronectin-treated plates, compared to *Nf1^−/−^* cells [57].

There is also data to suggest that loss of neurofibromin leads to epithelial-mesenchymal transition (EMT). EMT is implicated in tumorigenesis and cancer metastasis. Immunohistochemical analysis and real-time quantitative reverse transcription polymerase chain reaction showed increased expression of EMT-related transcription factors including Snail, Slug, Twist, ZEB1 and ZEB2 in NF1-associated neurofibroma specimens and NF1-derived Schwann cells. Knockdown of *NF1* with siRNA induced the expression of these transcription factors in normal human Schwann cells as well as epithelial-like breast cancer cell lines [58].

More recently, loss of *NF1* has been reported to promote carcinogenesis by activating heat shock factor 1 (HSF1), the master transcriptional regulator of the heat shock response. Knockout of *NF1* in MEFs triggered activation of HSF1, increasing HSF1 levels. This resulted in *Nf1^−/−^* cells becoming tolerant to proteotoxic stress with proteasome inhibitors and HSP90 inhibitor. This activation of HSF1 relied on dysregulated MAPK signaling. HSF1, in turn, supported MAPK signaling. In *NPcis*^+/−^ mouse models where *Trp53* and *Nf1* genes are disrupted on the same chromosome to develop soft tissue sarcomas resembling human MPNSTs, *Hsf1* knockout impeded NF1-associated carcinogenesis by attenuating oncogenic RAS/MAPK signaling. In cell lines from human malignant peripheral nerve sheath tumors (MPNSTs) driven by *NF1* loss and in surgically excised human MPNSTs, HSF1 was also overexpressed and activated or phosphorylated [59].

### Tumours associated with NF1

Individuals with NF1 are predisposed to developing both benign and malignant tumours throughout life. The risk of malignancy is increased 2.5 to fourfold in NF1 compared to the general population [8, 60]. Average life expectancy is reduced by 10-15 years, with cancer being the most common cause of death [2].

The tumour types individuals with NF1 are at increased risk of developing include both nervous system and non-nervous system tumours. The characteristics of the more common NF1-associated tumours are listed in Table 3. Accurate estimation of the relative frequencies of the various tumour types is challenging, as different studies based on hospital data may overestimate the frequency of specific tumours compared to population-based studies. This partly accounts for the wide range of prevalence or incidence figures reported in the literature for various tumours.

Malignant peripheral nerve sheath tumours (MPNSTs), previously referred to as neurofibrosarcomas, are a major cause of morbidity and mortality in NF1. MPNSTs typically arise from malignant transformation of plexiform neurofibromas, and occasionally spinal nerve root or subcutaneous neurofibromas. In NF1 the lifetime risk of developing MPNST is 8-13%, with estimated annual incidence at 0.16%, compared to 0.001% in the general population [9, 61, 62].

There is a wide range of other NF1-associated tumours including optic pathway gliomas (OPGs), rhabdomyosarcomas, neuroblastomas and juvenile myelomonocytic leukaemias (JMML) in the paediatric setting, as well as gastrointestinal stromal tumour (GIST), phaeochromocytomas and carcinoid tumours in adults. OPGs, like MPNSTs, may occur in both children and adults [9, 61, 62]. More recently, an increased risk of breast cancer among women with NF1 has also been reported [63, 64]. Breast cancer in NF1 patients appears to have an aggressive phenotype in the two reported case series [65, 66].

NF1 patients are also at an increased risk of developing radiation-induced malignancies. In a study of NF1 patients treated with radiotherapy for optic glioma, the relative risk of second CNS tumour was 3.04 (95% CI, 1.29 to 7.15) [67]. Hence radiotherapy should be avoided in children with NF1, unless it is absolutely essential. In mouse model studies, *Nf1^+/−^* mice subjected to irradiation developed in-field tumours associated with NF1 such as phaeochromocytomas, as well as typical second malignant neoplasms such as sarcomas and breast cancers [68-70]. This may be related to upregulated, perturbed cell cycle and DNA repair pathways with *NF1*-haploinsufficiency, as observed in human lymphoblastoid cell lines from NF1-affected and normal individuals, as well as in lymphocytes from wildtype and *Nf1^+/−^* mice. Activation of DNA damage response (DDR) genes can paradoxically trigger oncogene-induced DNA damage and genomic instability, resulting in carcinogenesis [71, 72]. Interestingly, somatic monoallelic loss of *NF1* and *TP53* in the adjacent allele was observed in radiation-induced malignancies arising in both wildtype and *Nf1^+/−^* mice in one study [68]. *NF1* loss appears to be a critical event in mutagen-induced malignancies beyond the classical NF1-associated tumour types.

**Table 3:** Tumours associated with NF1 syndrome

Tumour Type Associated with NF1	Age category	Frequency	Mechanism(s)	Differences compared to sporadic tumours	References
Malignant peripheral nerve sheath tumour (MPNST)	Adult, Paediatric	Lifetime risk 8-13%	LOH of *NF1*, mutation in *TP53*, copy number alterations, including deletion of *CDKN2A*, loss of *PTEN*	Earlier onset; central rather than peripheral location	[2, 9, 61, 62, 73-80]
Optic pathway glioma (OPG)	Adult (usually young), Paediatric	Incidence 1.5%-7.5% (Patil) prevalence 5-25%	*LOH* of *NF1*, mutation in *TP53*, deletion of *CDKN2A*	Earlier onset; anterior rather than posterior optic pathway	[9, 61, 62]
Rhabdomyosarcoma	Paediatric	Prevalence 1.4-6%	unknown	Earlier onset; urinary tract rather than head and neck	[9, 61, 81]
Neuroblastoma	Paediatric	Unknown	LOH of *NF1*, amplification of *MYCN*, deletion of 1p36		[9, 82, 83]
Juvenile myelomonocytic leukaemia (JMML)	Paediatric	Lifetime risk 200-fold increased	LOH of *NF1*, or compound heterozygous microlesions.		[62, 84-86]
Gastrointestinal Stromal Tumours (GISTs)	Adult	Lifetime risk 6%	LOH of *NF1*, some copy number alterations	Small intestine and multiple rather than gastric origin; lack of response to imatinib with lack of *KIT* and *PDGFRA* mutations	[9, 61, 87, 88]
Phaeochromocytoma	Adult	Prevalence 1%	LOH of *NF1*	Earlier onset; occasionally bilateral or extradrenal	[9, 61, 62, 89-91]
Carcinoid	Adult	Prevalence 1%	LOH of *NF1*	Earlier onset; periampullary rather than small intestine	[61, 62, 92]
Breast Cancer	Adult	Standardised incidence ratio of 3.5 to 5.2	Unknown	Earlier onset; possibly more aggressive	[63-66]

### Somatic NF1 Aberrations in Sporadic Tumours and Effects of NF1 Deficiency

**Figure 2:**
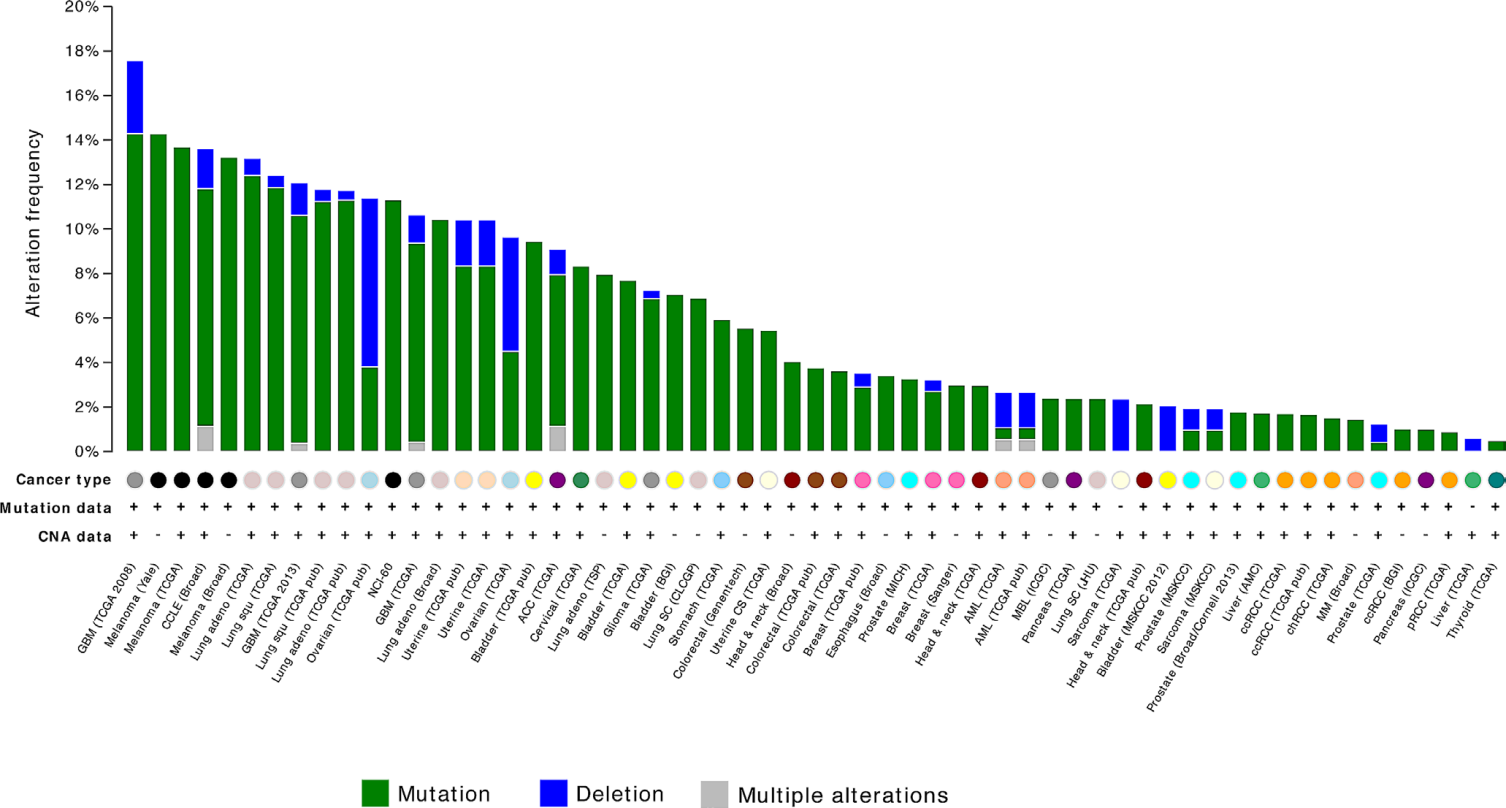
Frequency of NF1 mutation and homozygous deletion in human neoplasms (Source: The cBio Cancer genomics Portal; http://www.cbioportal.org)

With the recent cancer genome sequencing projects, the heterogeneity of cancer genomes has been unraveled. Somatic *NF1* aberrations are increasingly reported in various sporadic tumours, including brain, lung, breast, ovarian tumours as well melanomas and leukemias (Figure 2). This is particularly relevant with the advent of novel molecular therapies which can potentially be targeted at aberrations in the *NF1* pathway. Improved understanding of the mechanisms of carcinogenesis is critical for the optimisation of these targeted therapies.

### Brain Tumours

In glioblastoma multiforme (GBM), *NF1* is one of the most frequently mutated or deleted genes. The prevalence of *NF1* somatic mutations in sporadic GBMs was initially estimated to be approximately 15%, with a subsequent study by The Cancer Genome Atlas (TCGA) network reporting aberrations in at least 23% (47 out of 206) of human GBM samples when both *NF1* inactivating mutations and deletions (including heterozygous deletions) were analysed [93, 94]. However, when only mutations and homozygous deletions are considered, the frequency of alterations ranges from 12.1 to 17.6% [94, 95].

Data from mouse models support the importance of *NF1* as a glioblastoma suppressor gene. Inactivation of *TP53* and *PTEN* may cooperate with *NF1* loss to induce the malignant transformation [96]. Haploinsufficiency for the *NF1* tumour suppressor may have functional consequences, such as increased astrocyte proliferation and augmentation of angiogenesis in *Nf1^+/−^* heterozygous mouse models [97, 98]. Integrated genomic analysis of the TCGA data identified GBMs with *NF1* and *PTEN* alterations to have a distinct mesenchymal-like expression profile. This mesenchymal subtype was characterised by the expression of mesenchymal markers such as *CHI3L1* (also known as *YKL40*) and *MET*, as well as astrocytic markers (*CD44, MERTK*), reflecting the epithelial-to-mesenchymal transition. There was also high expression of genes in the tumour necrosis factor (TNF) and NK-ĸB pathway, related to the greater necrosis and associated inflammatory response in this subtype [99, 100].

### Melanoma

Loss of *NF1* in malignant melanoma cell lines was reported soon after discovery of the *NF1* gene in the early 1990s [101, 102], but it was only recently that comprehensive genomic characterization of melanomas was performed. Melanomas may be classified into 3 major classes: 1) sun-shielded melanomas with wild type *BRAF* and *NRAS* which have low mutation load but high number of copy gains, 2) sun-exposed melanomas with *BRAF* or *NRAS* mutations and 3) sun-exposed melanomas with wild-type *BRAF* and *NRAS*, few copy number alterations but high mutation load. The last subtype of melanoma was typically associated with more advanced age, and 30% of melanomas from this class (10/33 samples) carried deleterious *NF1* mutations. *TP53, ARID2* and *PTPRK* were frequently mutated in these melanomas, suggesting that inactivation of tumour suppressors contribute to the pathogenesis of these *BRAF* and *NRAS* independent tumours [103, 104]. The overall frequency of *NF1* mutations is estimated at 14% of cutaneous melanomas, with total of 475 specimens analysed so far in 3 separate studies (Figure 2) [103, 105].

Somatic *NF1* mutations have also been reported in melanoma specimens harboring *BRAF* mutations [105, 106]. In a mouse model study, *NF1* mutations cooperate with *BRAF* mutations in the pathogenesis of melanomas by preventing oncogene-induced senescence [106]. Loss of neurofibromin expression and *NF1* loss-of-function mutations have been reported in melanomas from patients with *de novo* as well as acquired resistance to BRAF inhibitors [106, 107]. A pooled RNA interference screen targeting >16,500 genes in a BRAF inhibitor-sensitive melanoma cell line identified *NF1* as the highest ranking gene whose knockdown abrogated the growth inhibitory effects of PLX4720, a BRAF inhibitor [107]. *NF1*-mutant melanomas are unlikely to respond to standard BRAF-targeted therapies but may benefit from drugs targeting the MEK and PI3K pathway instead. In mice injected with *BRAF/NF1*-mutated melanoma cells, there was resistance to vemurafenib, a BRAF inhibitor. In contrast, there was greater sensitivity to MEK inhibitor PD0325901, PI3K inhibitor GDC-0941 and rapamycin, an mTOR inhibitor. Importantly, rapamycin synergized with PD0325901, resulting in tumour regression in the allografts [106].

### Lung Cancer

Whole exome or genome sequencing of primary lung adenocarcinomas identified *NF1* as one of the most frequently mutated genes, with an estimated frequency of 11-12% [108, 109]. The clinical significance of *NF1* mutations in the lung adenocarcinoma sequencing studies is not reported, but reduced *NF1* mRNA expression was recently found to confer both intrinsic and acquired resistance to EGFR inhibitors in another recently reported study. However, somatic *NF1* mutations were not found in the specimens from these patients with resistant tumours (more details in section on challenges of molecular diagnosis of NF1 and *NF1* somatic aberrations) [110].

Approximately 12% of squamous cell lung cancers have alterations in *NF1*, according to a recently published TCGA study on squamous cell carcinomas. mRNA expression profiling identified 4 distinct subtypes of squamous cell lung cancers – classical, primitive, basal and secretory expression subtypes. The basal expression subtype of squamous cell lung carcinoma characteristically showed alterations in *NF1* [111].

There is less data on small cell lung cancer, but the frequency of mutations in *NF1* was reported as 2.4% and 6.9% in two separate studies of a smaller scale [112, 113].

### Ovarian Carcinoma

The importance of *NF1* in ovarian cancer was first reported by Sangha et al [114]. Initial genome-wide screen of DNA copy number alterations (CNAs) identified apparent *NF1* homozygous deletions in 2 out of 36 primary ovarian serous carcinomas. This led to the discovery that 6 out of 18 ovarian carcinoma-derived cell lines had markedly reduced or lacked expression of NF1 protein, with 5 of the 6 cell lines harbouring *NF1* mutations. Alterations in *NF1*, including splicing mutations and homozygous deletions, were identified in 22% (9/41) of the primary ovarian serous carcinomas studied. There was evidence of Ras pathway activation in these tumours and cell lines with *NF1* defects, in the absence of *KRAS* or *BRAF* mutations. *NF1* appears to cooperate with *TP53* mutations which are present in virtually all ovarian serous carcinomas, in carcinogenesis [114].

In the large scale integrated genomic analyses of 489 high grade serous ovarian carcinomas by the TCGA cooperative group, *NF1* has been recognized as one of the most frequently altered genes, with aberrrations in 12% of the cases (8% homozygous deletions, 4% mutations) [115]. These alterations affect signaling in the PI3K/Ras pathway, and may have therapeutic implications as discussed later in this review.

### Breast Tumours

Although a computational biology study on gene expression datasets had previously reported associations between the activity levels of regulatory pathways linked with *NF1* to clinical outcome in breast cancer [116], the importance of *NF1* in the pathogenesis of breast cancer was not investigated further until recently. Absence of neurofibromin protein and lack of expression of *NF1* mRNA type 1 isoform have been reported in the highly aggressive human breast cancer MDA-MB-231 cell line which is resistant to endocrine and cytotoxic agents. This was associated with accumulation of phosphorylated MAPK and activated Ras [117]. More recently, this Claudin-low subtype cell line was found to harbour *NF1* mutation [118]. The Cancer Genome Project led by the Sanger Institute and The Cancer Genome Atlas (TCGA) projects reported *NF1* mutations in approximately 3% of the breast cancers sequenced. Proportionally more *NF1* mutations were found in luminal or ER+HER2- subtypes, although they were also present in selected HER2-overexpressing and triple negative or basal tumours [119, 120]. This may have therapeutic implications, given that knockdown of *NF1* in MCF7 cells conferred resistance to tamoxifen in a genome-wide functional study [121].

*NF1* has also been implicated as a breast cancer driver in a recent mouse model study. *Chaos3* mice, which are engineered with a point mutation in the minichromosome maintenance 4 (*Mcm4*) gene, are highly unstable genomically, leading to the development of mammary tumours which resemble human breast cancers [122]. *NF1* was found to be deleted in nearly all the mammary tumours from these mouse models. This led to re-examination of the TCGA data. 27.7% of human breast cancers in the TCGA project were subsequently found to harbour *NF1* aberrations, majority of which were heterozygous deletions. Over 40% of HER2-overexpressing and basal subtypes showed these aberrations. This highlights the importance of investigating for the loss of *NF1*, in addition to the mutations [122].

Loss of heterozygosity of *NF1* has been detected in radiation-induced breast cancers from patients without NF1 syndrome. The monoallelic loss of *NF1* is likely to increase the potential for cooperating with other pathways such as *TP53* pathways to promote cellular proliferation and carcinogenesis [68]. Loss of *NF1* gene has also been reported in malignant phyllodes tumour of the breast [123].

### Haematological Malignancies

*NF1* was previously implicated as one of the important drivers in certain sporadic haematological malignancies. Myeloid malignanices frequently harbor mutations in the Ras pathway. It is likely that *NRAS/KRAS/NF1* aberrations cooperate with mutations in transcription factors and genes that regulate the epigenome in complex events leading to the development of AML [124]. In earlier studies, *NF1* mutations were reported in up to 7% of acute myeloid leukemia (AML) cases, while 12% of 95 cases studied had copy number alterations in *NF1* with mainly heterozygous deletions. Complete absence of *NF1* expression was reported in 7% of adult AML, and this was associated with increased Ras-bound GTP [125]. In another study on a subset of AML with *CBFB–MYH11* rearrangements, 16% of cases showed deletion of *NF1* [126]. However, two recent large scale studies suggested that *NF1* aberrations are not as frequent in de novo AML, although it may occur as a secondary event in disease progression [127, 128]. After taking into account the size of the gene in the test for significantly mutated genes, *NF1* is not one of the significantly mutated genes in AML, with the gene altered in 2.7% of 187 cases [128].

Limited data suggests the frequency of *NF1* alterations in myelodysplastic syndrome (MDS) varies from 0% to 9% [129, 130]. Recurrent cryptic alterations or deletions of the *NF1* locus have been detected in 3 out of 35 patients in one of the studies [129]. The frequency of *NF1* mutations in sporadic acute lymphoid leukemia (ALL) was recently reported as 3-8% [131, 132]. The prevalence of *NF1* aberrations in other haematological malignancies such as multiple myeloma is currently unclear.

### Colorectal Carcinoma

Data on the nature and the frequency of *NF1* aberrations in colorectal carcinoma vary widely. After the initial report by Li et al that 1 out of 22 sporadic colon adenocarcinomas (4.5%) harboured the amino acid substitution altering Lys-1423 in the *NF1* GRD [133], loss of heterozygosity (LOH) involving the *NF1* gene in 14-57% of colorectal carcinomas was reported in two small studies [134, 135]. In addition to *NF1* missense mutations, Ahlquist et al also found duplication of the whole *NF1* gene or parts of it in 4 out of 24 specimens (17%) [136]. Nine out of ten *NF1* mutations detected in this study occurred in introns likely involved in exon splicing. Notably, 8 of these 10 carcinomas showed microsatellite instability [136]. In contrast, *NF1* was found to be altered in approximately 3.8-5.6% of colorectal carcinomas in two recent next generation sequencing studies [137, 138].

### Other Sporadic Tumours

As displayed in Figure 2; there are several other tumours in which *NF1* aberrations have been reported.

Aberrations of *NF1* have also been reported in sporadic soft tissue sarcomas. Up to 10.5% of myxofibrosarcomas and 8% of pleomorphic liposarcomas harbor *NF1* mutations [139, 140]. In a study on embryonal rhabdomyosarcoma, loss of *NF1* occurred in 35%(9/26) of tumours (heterozygous or homozygous deletion of *NF1* or heterozygous chromosomal loss), and were mutually exclusive with *Ras* mutations, suggesting *NF1* loss as an alternative and potentially common driver of Ras activation in this major subtype of soft tissue sarcoma in young children [140].

A few studies reported somatic *NF1* aberrations or inactivation in 26-41% of sporadic phaeochromocytomas from individuals without NF1. In keeping with the observation that NF1 individuals are at increased risk of developing phaeochromocytomas, these findings suggest that loss of *NF1* function is a crucial event in the pathogenesis of both sporadic and NF1-associated phaeochromocytomas [91, 141, 142].

### Challenges of molecular diagnosis of NF1 and detection of NF1 somatic aberrations

The diagnosis of NF1 syndrome is usually established clinically in individuals with constitutional features of the syndrome. Germline *NF1* testing is reserved mainly for equivocal cases, for prenatal diagnosis and in the research setting. Detection of *NF1* mutations or deletions can be highly challenging due to several factors. *NF1* is one of the largest genes, with 60 exons spanning over 350kb of DNA. The gene also has one of the highest mutation rates, with up to half of the mutations being novel mutations. In addition to the myriad of possible lesions with more than 1,200 different germline mutations reported so far (source: The Human Gene Mutation Database; http://www.hgmd.org) and the lack of mutation hotspots, the presence of several pseudogenes can complicate the molecular diagnosis further [11, 143-147]. A multi-step protocol involving analysis of genomic DNA and mRNA with RT-PCR, direct sequencing, multiplex ligation-dependent probe amplification (MLPA), and previously using also microsatellite marker analysis and FISH, was required to identify up to 95% of pathogenic mutations in individuals fulfilling the clinical NIH diagnostic criteria [148-150]. Analysis of RNA is essential as splicing mutations may be present in more than 20% of individuals with NF1 syndrome [144, 149, 150], and may be located deep in introns which may be missed when only exons are studied.

Given the potential difficulties of detecting the pathogenic mutation in individuals with clinical features of NF1, the identification of somatic *NF1* aberrations in sporadic tumours can also pose a significant challenge. It is possible that the frequency of somatic *NF1* alterations in various tumours is higher than what is currently recognized.

Although next generation sequencing (NGS) may be less laborious than direct sequencing, there are also limitations with NGS techniques. Decreased specificity of the capture probes may lead to the capture and enrichment of off-target sequences, including those from pseudogenes and closely related genes [143]. Exome sequencing alone may not detect splicing mutations or gene rearrangements. Whole genome sequencing combined with transcriptome analysis may be superior, but there are limitations to its applicability in the clinical setting currently due to the general requirement for fresh frozen tissue, complexity of data analysis and cost.

### Downregulation of NF1 and neurofibromin via other mechanisms

Epigenetic factors, such as gene silencing by microRNAs and DNA methylation, may also influence the expression of *NF1* and neurofibromin, as described below.

microRNAs are endogenous, small noncoding RNAs which can influence their target gene expression post-transcription. Downregulation of *NF1* by microRNA-193b, which is overexpressed in sporadic head and neck squamous cell carcinomas (HNSCC), led to activation of ERK and resulted in tumour progression. Survival outcomes in HNSCC patients whose tumours expressed high levels of miR-193b were inferior compared to patients with low miR-193b expression. Knockdown of miR-193b in HNSCC cells increased *NF1* transcript and protein expression levels, decreased ERK phosphorylation with reduction in cell viability, migration, invasion and tumour formation [151].

There is limited data on methylation changes, but methylation of *NF1* was recently found to be the cause of a somatic second-hit inactivation in pilocytic astrocytoma from a patient with NF1 [152].

Excessive proteasomal degradation of neurofibromin can also result in deficiency of this critical tumour suppressor protein [153]. The ubiquitin ligase complex which controls both the regulated destruction and pathogenic destabilisation of neurofibromin was recently identified in glioblastomas as a Cullin 3(Cul3)/kelch repeat and BTB domain-containing 7 complex. Inhibition of Cul3 with Cul3-specific shRNAs suppressed Ras/ERK signaling; agents aimed at blocking neurofibromin destruction may be a potential therapeutic strategy for further development [154].

Given that the expression of *NF1* may be influenced by epigenetic factors, microRNAs [155] and proteasomal degradation [153, 154], a proteomics-based approach may help to detect deficiency of neurofibromin. The utility of immunohistochemical staining of neurofibromin has not been fully explored. Complete absence of neurofibromin staining on immunohistochemistry was found in 15-18% of melanomas [106]. However, quantitation of protein expression correlating with treatment outcomes has not been well studied. This is also complicated by the fact that current antibodies available may not be able to distinguish between the normal and mutant neurofibromin protein. Functional studies of “mutant neurofibromin” will be challenging with the huge protein size and myriad abnormalities possible.

The challenges of elucidating the mechanisms of *NF1* deficiency are demonstrated in the recent study on reduced *NF1* expression as a driver of resistance to EGFR inhibitor in lung cancer. *NF1* mRNA expression was reduced in EGFR TKI-resistant lung cancer specimens, but somatic mutations and methylation changes involving *NF1* were not detected. To account for the downregulation of *NF1* mRNA, immunohistochemistry using multiple antibodies was performed, but none of them demonstrated adequate specificity to detect neurofibromin in human lung tissue [110].

### Therapeutic Strategies for NF1 and NF1-associated/deficient malignancies

#### Management Options for NF1 syndrome and neurofibromas

The management of individuals with NF1 consists mainly of surgical resection of neurofibromas when they cause discomfort or impingement of neighbouring structures such as nerves or spinal cord. There is an unmet need for novel molecular therapies to treat the systemic manifestations in NF1.

Early clinical trials using thalidomide, 13-cisretinoic acid (CRA) or interferon α-2a to target angiogenesis and differentiation in NF1 patients with plexiform neurofibromas induced at best a minor response in a minority of patients [156, 157]. Early phase trials using pirfenidone, an antifibrotic agent drug which targets the stromal contributions, showed similar limited activity in plexiform neurofibromas in adults and children [158, 159].

Since Ras is overactivated with dysfunction of *NF1*, subsequent NF1 trials focused on inhibition of Ras. Farnesylation and geranylgeranylation of Ras proteins is essential for translocation to the cell membrane with subsequent activation of the Ras pathway. The activity of tipifarnib, a farnesyl transferase inhibitor, was reported in a phase 1 trial on children with solid tumours or NF1 and plexiform neurofibromas. Stable disease was the best response; no significant regressions were observed [160]. More recently, in a phase 2 placebo-controlled study on children and young adults with NF1 and progressive plexiform neurofibromas, tipifarnib did not prolong the time to progression compared to placebo [161]. Similarly, results from a phase 2 study using sirolimus (rapamycin), an mTOR inhibitor, in NF1 patients with plexiform neurofibroma, did not report any regression of the lesions [162]. Clinical trials using everolimus, a newer generation mTOR inhibitor and other therapies are in progress (http://www.clinicaltrials.gov). The MEK inhibitor PD0325901 was effective in shrinking plexiform neurofibromas in more than 80% of genetically engineered mice, but data on clinical activity in human subjects is awaited [163].

Pegylated interferon-α-2b, which has antiproliferative, antiangiogenic and immunomodulatory properties, induced minor response in 29% of young patients with plexiform neurofibromas in a phase I trial [164]. Tumour stabilization or prevention of new lesions may be a more realistic endpoint as dramatic regression of established “benign” tumours is less likely. Although neurofibromas may show LOH in a subset of Schwann cells, the mode of pathogenesis is different from that of malignant tumours [165]. However, imatinib mesylate, an oral kinase inhibitor targeting c-kit and PDGFRβ, was recently reported to decrease plexiform neurofibromas by 20% or more in 6 out of 36 NF1 patients in a phase 2 trial. This effect may partially be related to targeting cellular phosphor-signalling cascades [166-168]. In contrast, sorafenib which targets c-kit and PDGFRβ as well as RAF, VEGFR2, was poorly tolerated and did not show any tumour response in a phase 1 trial on children with NF1 and plexiform neurofibromas [169]. The clinical efficacy of these compounds in treating neurofibromas remains to be tested in larger clinical trials.

#### Potential therapeutic strategies for NF1-deficient malignancies

**Figure 3:**
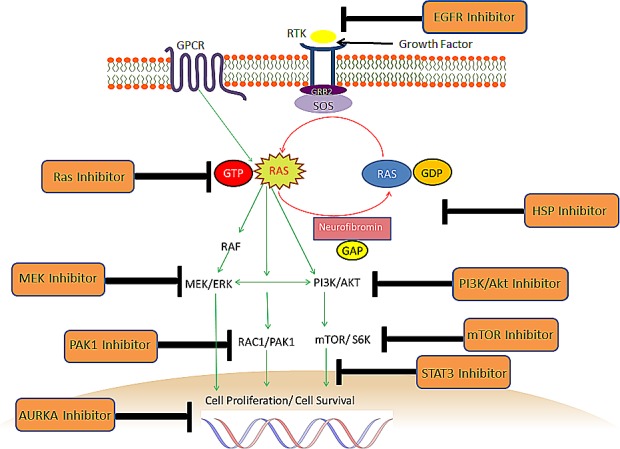
Potential therapeutic strategies for NF1-deficient malignancies The molecular therapies above have been tested in the preclinical setting, largely for MPNSTs. There is also data on some of the inhibitors for neurofibromin-deficient breast cancer, glioblastoma, AML, soft tissue sarcoma, lung cancer and melanoma. Combination therapy targeting more than one checkpoint may be required for optimal inhibition.

Data on the efficacy of molecular therapies in NF1-deficient malignancies is currently limited to results from preclinical studies (Figure 3). Much of this research has been conducted on models of MPNST derived from NF1 patients. This is set to change with emerging clinical trials where the molecular therapy is matched to the genomic profile of each individual's tumour. A one-size fits all approach may not always deliver an optimal outcome. For instance, although imatinib is standard-of-care for most patients with sporadic GIST, *KIT/PDGFRA* mutations are uncommon in GISTs arising in NF1 individuals, so response to imatinib is poor in these patients [88].

There is preclinical data to support the activity of MEK inhibitors, Ras inhibitor farnesylthiosalicylic acid, sirolimus, everolimus and PI3K/Akt/mTOR inhibitors in MPNST cell lines or xenografts derived from NF1 patients [163, 170-174] (Figure 3). The addition of erlotinib, an epithelial growth factor receptor (EGFR) inhibitor to everolimus, inhibited growth and induced apoptosis further in 4 NF1-derived and 1 sporadic MPNST cell lines as well as the STS26T sporadic MPNST xenograft [172]. EGFR expression is present in most MPNST cell lines, and the EGFR signaling pathways were found to be associated with tumorigenesis in the Nf1:p53 mouse tumor model [172, 175].

Signal transducer and activator of transcription-3 (STAT3) is a potential target for treating NF1-associated or NF1-deficient cancers, as STAT3 is activated downstream in the PI3K/mTOR pathway. The natural product cucurbitacin-I, a potent STAT3 inhibitor, was found to inhibit the growth of NF1-deficient MPNST cells in vitro and in vivo in xenografts [176].

Since heat shock factor is activated with loss of NF1, it is not surprising that the addition of HSP90 inhibitor IPI-504, to rapamycin, led to synergistic activity with damage of endoplasmic reticulum and mitochondria in NF1-deficient MPNST mouse models [177].

More recently, integrative transcriptome analyses have identified Aurorakinase A(*AURKA*) as a potential therapeutic target. *AURKA* was overexpressed and amplified in NF1-related MPNST, but not neurofibromas. MLN8237, an AURKA selective inhibitor, was effective in stabilizing tumour volume and prolonged survival of mice with MPNST xenografts [178].

Inhibitors of PAK1, a downstream effector in the Ras pathway, have also been reported to suppress the growth of *NF1*-deficient MPNST cells as well as neurofibromin-deficient human breast cancer (MDA-MB-231) xenografts in mice. There is evidence that many tumours, including breast cancers, are addicted to abnormal activation of PAK1, a Ser/Thr kinase which in turn stimulates cyclin D1, for their growth [179, 180].

In sporadic tumours harbouring *NF1* aberrations, MEK inhibitors have been found to be effective in treating neurofibromin-deficient sporadic glioblastoma cell lines, *NF1*-deficient AMLs and *NF1*-deleted soft tissue sarcomas in mouse models [181-183]. Following the discovery that *NF1* deficiency confers intrinsic and acquired resistance to EGFR inhibitor in lung cancer, treatment of neurofibromin-deficient lung cancers *in vitro* and in xenografts with MEK inhibitory drugs (AZD-6244, CI-1040 and PD0325901) restored sensitivity to erlotinib when given in combination [110].

Combination therapies targeting more than one checkpoint in the cell proliferation pathway, such as blocking both the PI3K/mTOR and MEK pathways in the allografts of *NF1/BRAF*-mutated melanomas and dual EGFR, MEK inhibition concurrently in TKI-resistant NF1-deficient lung adenocarcinomas, may be superior to monotherapy [106, 110]. Inhibiting a single checkpoint may lead to activation of compensatory negative feedback pathways.

Future strategies may include inhibition of excessive destruction of neurofibromin and other epigenetic therapies. In *Nf2*-mutant Schwann cells, inhibition of SIRT2, a class III histone deacetylase, triggered necrosis [184]. The role of HDAC inhibitors, which may decrease Akt phosphorylation, has not been fully explored for *Nf1*-mutant cells. Inhibition of LIM kinase in the Rho-ROCK-LIMK-cofilin pathway regulated by neurofibromin is another potential strategy. In *Nf1^−/−^* MEFs, novel LIMK inhibitors blocked the phosphorylation of cofilin, resulting in actin severance and inhibition of cell migration and growth [185]. The utility of these drugs in NF1-deficient tumours may be worth investigating, especially in combination with Ras or AURKA inhibitors, which may have synergistic effects [185, 186]. Improved understanding of the biology of *NF1* and neurofibromin in normal cells and cancer is critical for the development of novel treatment strategies.

## CONCLUSIONS

*NF1* and neurofibromin play critical roles in tumour suppression. The frequency of somatic *NF1* aberrations in sporadic tumours is increasingly recognized. These alterations are associated with distinct subtypes in certain cancers, and may be associated with poorer treatment outcomes. Significant challenges remain in unravelling the complexity of the large *NF1* gene and its product neurofibromin. Improved molecular diagnosis techniques are essential for detecting these aberrations. There is also an unmet need to develop novel systemic therapies for treating *NF1*-deficient tumours.
